# *Coe1* in *Beta vulgaris* L. Has a *Tnp2*-Domain DNA Transposase Gene within Putative LTRs and Other Retroelement-Like Features

**DOI:** 10.1155/2008/360874

**Published:** 2008-06-11

**Authors:** David Kuykendall, Jonathan Shao, Kenneth Trimmer

**Affiliations:** Molecular Plant Pathology Laboratory, ARS, USDA, Beltsville, MD 20705, USA

## Abstract

We describe discovery in *Beta vulgaris* L. of *Coe1*, a DNA transposase gene within putative long terminal repeats (LTRs), and other retrotransposon-like features including both a retroviral-like hypothetical gene and an Rvt2-domain reverse transcriptase pseudogene. The central DNA transposase gene encodes, in eight exons, a predicted 160-KDa protein producing BLAST alignments with *En/Spm*-type transposons. Except for a stop signal, another ORF encodes a *Ty1-copia*-like reverse transcriptase with amino acid sequence domain YVDDIIL. Outside apparent LTRs, an 8-mer nucleotide sequence motif CACTATAA, near or within inverted repeat sequences, is hypothetical extreme termini. A genome scan of *Arabidopsis thaliana* found another example of a *Tnp2*-domain transposase gene within an apparent LTR-retrotransposon on chromosome 4.

## 1. INTRODUCTION

 Since the discovery of transposable elements (TEs) in corn [1], DNA sequencing has revealed that genomes of eukaryotic organisms are largely comprised of evolutionarily significant TEs responsible for creation of considerable genetic diversity [2]. The movement of transposable elements is either autonomous or dependent on other elements. Classified according to mode of transposition, Class I TEs, or retrotransposons, are retroviral-type elements which may or may not have long terminal (direct) repeats (LTRs) [3]. Movement of Class I elements necessarily involves an RNA inter-mediate in what can appropriately be termed “replicative” transposition. Retrotransposons are transcribed into RNA, and then the reverse transcriptase and integrase make and insert a DNA copy at a secondary genomic location. Class II TEs are often called “DNA transposons,” but it is important to note that Class I retrotransposons are also comprised of DNA except during transposition. Transposases permit Class II transposable elements to move by a “cut and paste” process, first excising from one site and then reintegrating at another. DNA replication is not required. “Footprints,” telltale evidence for a previous DNA transposon insertion, result from imprecise excision. Class II transposons characteristically have relatively short inverted repeat sequences near their termini and an excision site at each end recognized by the transposase.

One of the first plant transposons McClintock described [1], *En1*, or the maize suppressor-mutator (*Spm*), is the original example of a “CACTA” class, or superfamily, of transposons. CACTA transposons were thought until recently to be found only in plants, but a similar element was discovered [4] in the genome of *Schistosoma mansoni*, the causative agent of schistosomiasis.

Evidence that retrotransposons account for much of the sugar beet (*Beta vulgaris* L.) genome was first obtained by Schmidt et al. [5], who described (1) repetitive DNA sequences in *Beta vulgaris* similar to long interspersed nuclear elements (LINEs), a type of retrotransposon without LTRs, and (2) other repetitive DNA sequences that resembled LTR retrotransposons of the *Ty1-copia* class. *Vulmar1*, a mariner-class transposon in *Beta vulgaris*, [6], 3 909 bp in length, has 32 bp terminal inverted repeats and carries a single ORF that encodes a transposase with a characteristic DDE signature motif in a single exon.

Our interest in repetitive DNA developed from our recent discovery of a number of LTRs and retrotransposon genes as well as a transposase gene in the region between two clusters of core plant genes on a 130 Kb sugar beet BAC [7, 8]. One gene cluster has an *NPR1*-class disease resistance-potentiating gene adjacent to another core plant gene whose predicted product has high similarity to a heat shock factor protein. The other cluster consists of a signal peptide calmodulin-binding protein kinase gene located near a CK1-class protein kinase gene. In this communication, we report the discovery in *Beta vulgaris* of *Coe1*, a Class II DNA transposase gene within putative LTRs and other features that are characteristic of Class I LTR-retrotransposons. Also, a genome scan of *Arabidopsis thaliana* found a similar arrangement of transposon and retro-element genes on chromosome 4.

## 2. MATERIALS AND METHODS

The identification of a sugar beet genome-derived bacterial artificial chromosome (BAC) carrying the *NPR1* disease resistance control gene has been previously described [7] as well as the basic methods used for DNA sequence analysis. In this study, analysis of the *NPR1* BAC was performed using LTR_STRUC (http://www.genetics.uga.edu/retrolab/data/LTR_STRUC.html), RepFind (http://zlab.bu.edu/repfind/form.html) analysis identified identical direct repeats. Etandem (http://bioweb.pasteur.fr/seqanal/interfaces/etandem.html) and Einverted (http://edukon.biologie.uni-konstanz.de/cgi-bin/emboss/einverted) were used to identify tandem and inverted repeats. EMBOSS [9] (http://emboss.sourceforge.net/) was also utilized to identify tandem repeats and inverted repeats. Repeats were also found using NCBI's BLASTprogram (http://www.ncbi.nlm.nih.gov/BLAST) by BLAST of a contig against itself using BLASTn. A sugar beet expressed sequence tag (EST) database (http://genomics.msu.edu/sugarbeet/blast.html) was employed for nucleotide and protein BLASTs in order to identify possible functional gene expression. Subsequent analyses of DNA sequence data were performed using Lasergene ver. 6 (DNASTAR, Madison, Wis). Multiple alignments were performed using MegAlign from the DNASTAR suite. Phylogenetic tree analysis was performed using Mega 4 software (http://www.megasoftware.net/).

A new multicopy direct tandem repeat (MDTR) within an intronic region within the *Coe1* transposase gene was identified by BLAST of the intron against the entire 130 kb BAC and plotting a diagram of repeat versus DNA base positions with respect to the BAC. In order to find the starting points of the repeats, a window which was of a constant length less than one repeat was used in a sliding window technique. The first base used in this window was the putative starting point of the first repeat in the MDTR, as determined by the BLAST output. This window was BLASTed against the whole intron. If BLAST found any repeated DNA using this template, the window was actually still within the repeated segment; therefore, the starting base of the first repeat was deduced to be further towards the 5th end. The window was then moved a few bases in the 5th direction and the amended sequence was subjected to another BLAST. This process continued until BLAST no longer identified repeats.

GenBank accession EF101866 provides annotation of the 130 Kb *NPR1*-carrying BAC derived from the sugar beet genome. Conserved microsynteny of four core plant genes was observed with other eudicots (Kuykendall et al., submitted).

To scan the *Arabidopsis thaliana* genome for a *Coe1*-like element(s), each chromosome was individually subjected to LTR_STRUC analysis, and then each putative LTR-retrotransposon element was examined for both a DNA transposase gene and a retrotransposon-like integrase or reverse transcriptase gene within its LTRs.

## 3. RESULTS AND DISCUSSION

**Figure 1:**
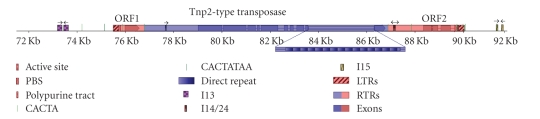
A schematic diagram of *Coe1*, a DNA transposon within an LTR retrotransposon. Inverted repeats are in checkerboard with arrows indicating direction. Dark green lines depict the 8-mer DNA sequence motif CACTATAA, whereas lighter green lines show the sequence motif CACTA. Heavily cross-hatched regions depict the putative LTRs. The lightly dotted blue and red regions show the composite element. Darker shaded red or blue boxes are exons of retroelement ORFs or of a central DNA transposase gene, respectively. A red-highlighted box with downward (backward) slanting lines depicts an apparent polymerase binding site (PBS). Lighter red or blue boxes, between exons, are introns. Repeating units of a light blue to dark blue gradient depicts repeating “MDTR” units within an intron of the DNA transposase gene (blue). A red box with horizontal lines depicts the apparent active site (save for a stop signal) of the predicted protein product encoded by retroelement integrase/reverse transcriptase. A box with upward (forward) slanting lines depicts a polypurine tract. Scale is in kilobases is shown with 2 Kb increments. Names of putative genes are shown above the respective ORFs in *Coe1*.

BLAST and LTR_STRUC analyses performed on an annotated 130 Kb *NPR1* gene-carrying sugar beet BAC (GenBank accession EF 101866) revealed the presence of *Coe1*, which appears to be a new and unique composite of Class I and Class II transposable elements. *Coe1* was chosen as its name to honor Dr. Gerald Coe who originally bred and developed a new U.S. hybrid sugar beet genotype, US H20 [10]. US H20 was the source of genomic DNA for a sugar beet Bacterial Artificial Chromosome (BAC) library 8 from which a BAC clone carrying the *NPR1* disease resistance control gene was recently identified [7]. Initially detected by LTR_STRUC analysis as a LTR-retrotransposon, *Coe1*, defined as 14.5 Kb by two putative 169 bp LTRs, has both an Rvt2-domain reverse transcriptase pseudogene and another retroviral-like hypothetical gene. However, a DNA transposase gene was found within its central region (Figure 1). In addition to *Coe1*, LTR_STRUC analysis performed on the 130-Kb *NPR1* BAC identified at least two other LTR-retrotransposons, briefly: (1) a *copia* or *Ty1*-like retroelement, *BvRTR1*, which has a reverse transcriptase with active site YVDDIIF; and (2) *BvRTR2*, a *gypsy* or *Ty*3-like retroelement with active site FIDDILI in its conserved *Rvt1* domain (unpublished). Precedence in the literature exists for similar yet considerably smaller repetitive DNA sequences from sugar beet largely uncharacterized except for genomic distribution.

The question then arises of whether the transposase gene of *Coe1* represents a Class II transposon inserted into a Class I LTR-retrotransposon. This is probably how *Coe1* originated. In any case, *Coe1* has salient features of a Class II DNA transposon within a Class I retrotransposon (Figure 1) as described below.

**Figure 2:**
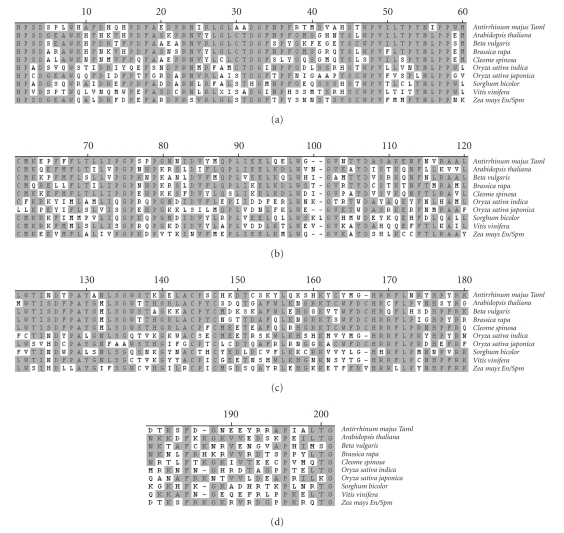
Amino acid residue alignment of a conserved region of the predicted product of *Beta vulgaris Coe1*'s DNA transposase gene with predicted products of DNA transposase genes from various other plants. Amino acids matching the consensus sequence are shaded. Numbers indicate cumulative amino acid positions. *Antirrhinum magus* Tam1 (X57297), *Arabidopsis thaliana* (BAB09502), *Beta vulgaris* (ABM55245), *Brassica rapa* (BAA85462), *Cleome spinosa* (ABD96944), *Oryza sativa indica* (CAH66091), *Oryza sativa japonica* (NP_001062816), *Sorghum bicolor* (AAM94290), *Vitis vinifera* (CAN82870), *Zea mays En/Spm* (AAA66266).

The *Tnp2*-domain transposase gene central to *Coe1* consists of eight exons. The transposasegene of *Coe1* has a predicted protein product that is evidently a CACTA superfamily DNA transposase as deduced from the results of BLAST amino acid sequence alignments of the predicted protein product with *En/Spm*-type DNA transposons (Figure 2).

Evidence for probable expression of *Coe1's* transposase gene, or at least a similar gene, were two sugar beet (expressed sequence tags)(ESTs) [11] whose nucleotide sequence aligned by BLAST with *Coe1*'s DNA transposase gene: CF542726 (*e* = 0.0) and BQ595658 (2*e* − 96).

**Figure 3:**
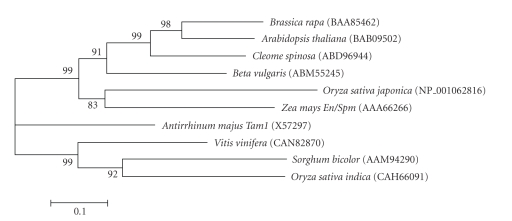
Phylogenetic tree, constructed by neighbor joining analysis, of the amino acid alignments shown in Figure 2. Genbank accession numbers of amino acid sequences follow the plant species name.

The *Coe1* DNA transposase gene is flanked by inverted repeats and a CACTA sequence motif (Figure 1). In addition to *Coe1*, the prototypical CACTA superfamily transposon *En/Spm* of corn, *Tam1* of snapdragon [12] and seven other *Tnp2*-domain transposons, from various plant species, were compared using MegAlign. Figure 2 shows the amino acid sequence alignments obtained with one of two conserved domains. Cluster analysis of these data (Figure 3), a neighbor joining analysis tree, indicates that the *Coe1* transposase falls into a group we designate as I subgroup A, with other plant *Tnp2*-domain transposases from *Arabidopsis thaliana* (BAB09502), *Cleome spinosa* (ABD969441), and *Brassica rapa* (BAA85462.1). Another subgroup of group I, B has *En/Spm* of *Zea mays* (AAA66266) and *Oryza sativa japonica* NP_001062816. In the amino acid sequence alignments of this particular conserved region (Figure 2), two other dissimilar groups (II and III) had the remaining four DNA transposases (Figure 3). The *En/Spm*-like superfamily of plant transposons, exemplified by Barbara McClintock's suppressor/mutator transposons of corn, has been named CACTA for the sequence motif recognized for excision. Conservation is well established over the taxonomic divide between eudicots, and monocots.

*Coe1* has a centrally located transposase gene, flanked downstream by a pair of imperfect 51 bp *I14* inverted repeat sequences (94% match) separated by only 10 bp, and upstream by another repeated sequence, *I24*, that aligns with *I14* with about 75% identity over 41 bp. These distal *I24/I14* inverted repeats are each flanked by a CACTA sequence motif.

*Coe1* has a total of three ORFs: a retroviral-like hypothetical gene ORF1, the *Tnp2*-domain transposase gene and an apparent *Rvt2*-domain reverse transcriptase pseudogene, ORF2. *Coe1* has putative long terminal repeats characteristic of LTR retrotransposons (Figure 1). These relatively short 169-bp direct repeats share only 96.4% identity. The 3.6% sequence divergence in the LTRs is consistent with possibility that the retroelement-like features of *Coe1* are no longer active. The 5th end of the *Coe1* positive strand has a pair of *I13* inverted repeats 173 bp apart with 76% match over 190 bp and an internal CACTATAA sequence motif. *I15* inverted repeats are found downstream of *Coe1* and these inverted repeats, near another CACTATAA sequence motif, are 52 bp apart with about 74% match over 51 bp.

ORF1, the first retroelement-like gene of *Coe1*, encodes, in a single exon, a hypothetical protein for which no significant BLAST alignment is currently found. The predicted protein product of ORF1 had initially produced a significant BLAST with a “polynucleotidyl transferase” but that accession has been withdrawn.

**Figure 4:**
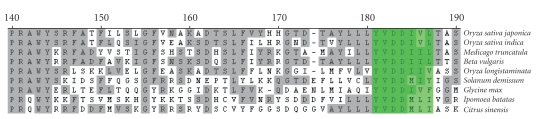
Amino acid residue alignment of a conserved region of the predicted product of *Beta vulgaris Coe1* ORF2, albeit interrupted with a stop codon, which otherwise would have contained an *Rvt2* domain with those of other retroelement reverse transcriptase genes from different plants. A conserved YVDDIIL active site is highlighted in green. *Beta vulgaris* (ABM55246), *Citrus sinensis* (CAJ09951), *Glycine max* (AAO73527), *Ipomoea batatas* (AAV88069), *Medicago truncatula* (ABE85780), *Oryza longistaminata* (AAB82754), *Oryza sativa indica* (CAH67061), *Oryza sativa japonica* (NP_001067469), *Solanum demissum* (AAT38758).

ORF2 produced only a relatively weak nucleotide BLAST alignment (2*e* − 25) to EST BI643401. ORF2 is apparently a pseudogene since a stop signal occurs in the sequence prior to that part of the sequence that would otherwise encode the active site of an Rvt2 domain reverse transcriptase. Although a sequencing error is possible, it is unlikely; therefore, it is reasonable to deduce from the sequence data that ORF2 of *Coe1* is a reverse transcriptase pseudogene. Disregarding the stop signal, the predicted protein product of the *Rvt2*-like gene of *Coe1* aligned well with other *Rvt2*-domain gene products (Figure 4). The *Coe1* *Rvt2* domain reverse transcriptase has a *Ty1-copia*-like YVDDIIL active site which is most highly conserved in comparison with that of the *Medicago truncatula* accession (Figure 4). Among the protein alignments performed, the hypothetical sugar beet *Rvt2*-domain containing gene product also showed higher similarity with retrotransposon-type reverse transcriptase proteins encoded by genes in two subspecies (*indica* and *japonica*) of *Oryza sativa* than with most others from a wide taxonomic range (Figure 4).

We recently performed a genome-wide scan or survey of the *Arabidopsis thaliana* genome looking specifically for a composite DNA transposon within LTR retrotransposon features similar to *Coe1*, and a similar single *Tnp2*-domain transposase gene flanked by putative LTRs and other retrotransposon-like features was identified, as described below.

**Figure 5:**
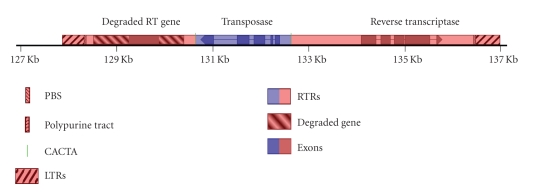
A schematic diagram of an *Arabidopsis Coe1*-like composite Class II transposon within a Class I LTR-retrotransposon. A central gene colored blue encodes a CACTA-superfamily transposase between retrotransposon genes colored red, all within retrotransposon LTRs. Light green lines depict the DNA sequence motif CACTA. Heavily dotted regions show LTRs. The lightly and darkly red and blue dotted box regions show the composite. A red box with downward (backward) slanting lines shows a RNA polymerase binding site (PBS). Darker red or blue boxes show exons. Dark red retrotransposon ORFs (dark) are interrupted by lighter red introns. A central DNA transposase gene is dark blue with light blue introns. A red box with upward (forward) slanting lines is a polypurine tract. Scale is in kilobases located below has 2 Kb increments. Names assigned putative genes are above their representations.

An *Arabidopsis thaliana* DNA transposase gene within putative LTRs and between LTR-retrotransposon genes is depicted in Figure 5. The apparent LTR-retrotransposon is 9 078 bp and has 506 bp and 471 bp LTR regions with about 90% identity. This element was found on BAC T26N6 from chromosome IV at 19.3 cM (accession AF07243). The first ORF (At4g04426) appears to be a highly degraded pseudogene of a reverse transcriptase, the central ORF (At4g04430) is a CACTA-class transposase gene, and the third ORF (At4g04440) has a predicted protein product with an Rvt2 reverse transcriptase domain similar to *Ty1-copia*-like retrotransposons. Alignment of a conserved region of the predicted product of the reverse transcriptase and a neighbor joining tree from cluster analysis (not shown) revealed a number of similar BLAST hits but none of these was very nearly identical to the predicted product of the *Arabidopsis* element. The finding of a *Coe1*-like element in *Arabidopsis* supports the concept that *Coe1* in *Beta vulgaris* is not a unique instance of a Class II transposon within a Class I retrotransposon.

**Figure 6:**
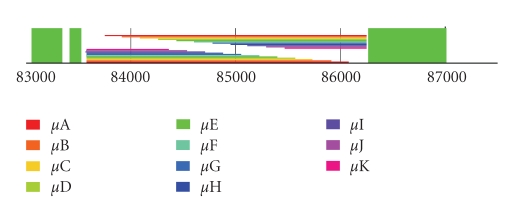
Diagram showing repeats within an intron of the *Coe1* transposase gene as initially identified by BLAST. Sets of multicolored lines represent lengths and position of a direct repeats. Green boxes depict the exons of the transposase gene. Scale is in base pairs in increments of 1000 base pairs.

Regarding the subject of *Coe1* in *Beta vulgaris*, it is noteworthy that within the transposase gene most of the DNA sequence of an intron, when BLAST against itself yielded multipl, relatively small regions of imperfect DNA sequence identity. These small regions aligned, albeit imprecisely, with each other. These results, when graphed, showed a distinct pattern (Figure 6) that suggested a whole series of multicopy direct tandem repeats (MDTRs).

Repeats of the apparent MDTR found by BLAST were of different lengths, but the first repeat in each set began at the same position, and the second repeat in each set ended at the same position. Each successive set of repeats was shorter by about the same number of bases. The intron was thus divisible into visually evident repeats (Figure 6).

Individual nonidentical MDTR repeats are 173 bp. Each repeated DNA sequence, aligned in MegAlign, gave the following consensus sequence: aggaacatgaaacccaaaaaagggct cgaaatggcttagtttctatcattttcattggctaagtgtattaaacttgcttagaatcata caaccatgtagtagaaagtttaaatgagtattttaggacttgcatgagccattagaactt gaaataggcatagaggtaggatta.

An intron within *Coe1*'s transposase gene was thus determined to have 15 complete 173 bp MDTR and a partial repeat at the 3′ end. The sequence of this MDTR-carrying intron did not yield any MegaBLAST hits against the NCBI database. One or more individual repeat(s) could have transposed into the intron followed by duplication(s), or possibly the entire series of multicopy tandem direct repeats transposed into the intron altogether. The latter possibility may be supported by the observation that the last repeat is partial, presumably a deletion.

How did *Coe1*, or an arrangement of transposable genes that resembles *Coe1*, originate? A DNA transposon could have moved into the middle of an LTR-retrotransposon. In other words, *Coe1*'s central transposase gene, flanked by inverted repeats and CACTA sequence motifs, could have transposed into an LTR-retrotransposon.

The CACTATAA sequence motif, located 16.3 Kb apart, outside the putative LTRs near large inverted repeats, could perhaps be extreme boundaries of *Coe1* instead of the two putative LTRs (Figure 1) separated by 14.5 Kb.

It is possible that the intact larger and more complex composite transposon could move about using DNA transposase. The LTRs are flanked by pairs of inverted repeat sequences that may be nonautonomous, miniature inverted [repeat] sequence transposable elements (MITEs) (Figure 1). MITEs, sometimes called “Class III transposons,” are dependent on DNA transposase. The 8-mer sequence motif CACTATAA flanks the *Coe1* LTRs near or within relatively large inverted repeats that are perhaps MITEs.

We hypothesize that originally a Class I LTR-retrotransposon inserted between pairs of MITEs. Then, a Class II DNA transposon moved into the middle of the LTR-retrotransposon, and voila, a composite of Class II and Class I elements resembling *Coe1*, at least conceptually. To summarize, *Coe1* has a *Tnp2*-domain transposase gene flanked by putative LTRs and between two retrotransposon-like genes, all within CACTATAAs near or within pairs of inverted repeats (Figure 1).

One may ask, “what possible selective advantage would a CACTA DNA transposon within an LTR retrotransposon have over a simple Class I or Class II element alone?” A combination of Class I and Class II features may offer little if any selective advantage, and thus such a composite might be unique. The finding of a similar gene arrangement in *Arabidopsis* provides a second example of a composite Class II transposon within a Class I retrotransposon.

A change in the expression of a gene when placed under the control of another can confer a selective advantage on the host plant. Increased fitness might also be characteristic of the host of a versatile element which can hypothetically transpose in either of two known mechanisms. Such versatility could facilitate more rapid genetic change due both to transposition and to subsequent blockage of gene conversion.

## 4. CONCLUSION

In conclusion, based on results of *in silico* analyses, *Coe1*, found in the sugar beet genome, can be viewed as an incipient or emerging CACTA super-family DNA transposon amalgamated within an LTR-retrotransposon. A similar arrangement of a central *Tnp2*-domain transposase gene within LTRs and between retrotransposon genes was found in chromosome 4 of *Arabidopsis thaliana* by a genome scan. 

More DNA sequencing of the sugar beet genome, either of larger stretches or of the complete genome, is likely to be needed in order to distinguish whether *Coe1* represents an evolutionarily significant gene arrangement or a mere coincidental merging of transposable genes.

In either case, as far we know, the two examples shown here of a Class II DNA transposon within a Class I retrotransposn are novel.

## References

[1] McClintock B (1950). The origin and behavior of mutable loci in maize. *Proceedings of the National Academy of Sciences of the United States of America*.

[2] Kidwell MG, Lisch D (1997). Transposable elements as sources of variation in animals and plants. *Proceedings of the National Academy of Sciences of the United States of America*.

[3] Casacuberta JM, Santiago N (2003). Plant LTR-retrotransposons and MITEs: control of transposition and impact on the evolution of plant genes and genomes. *Gene*.

[4] DeMarco R, Venancio TM, Verjovski-Almeida S (2006). SmTRC1, a novel *Schistosoma mansoni* DNA transposon, discloses new families of animal and fungi transposons belonging to the CACTA superfamily. *BMC Evolutionary Biology*.

[5] Schmidt T, Kubis S, Heslop-Harrison JS (1995). Analysis and chromosomal localization of retrotransposons in sugar beet (*Beta vulgaris* L.): LINEs and *Ty1-copia*-like elements as major components of the genome. *Chromosome Research*.

[6] Jacobs G, Dechyeva D, Menzel G, Dombrowski C, Schmidt T (2004). Molecular characterization of *Vulmar1*, a complete *mariner* transposon of sugar beet and diversity of *mariner*- and *En/Spm*-like sequences in the genus *Beta*. *Genome*.

[7] Kuykendall LD, Murphy TS, Shao J, McGrath JM (2007). Nucleotide sequence analyses of a sugar beet genomic *NPR1*-class disease resistance gene. *Journal of Sugar Beet Research*.

[8] McGrath JM, Shaw RS, de los Reyes BG, Weiland JJ (2004). Construction of a sugar beet BAC library from a hybrid that combines diverse traits. *Plant Molecular Biology Reporter*.

[9] Rice P, Longden I, Bleasby A (2000). EMBOSS: the European molecular biology open software suite (2000). *Trends in Genetics*.

[10] Coe GE, Hogaboam GJ (1971). Registration of US H20 sugarbeet. *Crop Science*.

[11] Herwig R, Schulz B, Weisshaar B (2002). Construction of a ‘unigene’ cDNA clone set by oligonucleotide fingerprinting allows access to 25 000 potential sugar beet genes. *The Plant Journal*.

[12] Nacken WKF, Piotrowiak R, Saedler H, Sommer H (1991). The transposable element *Tam1* from *Antirrhinum majus* shows structural homology to the maize transposon *En/Spm* and has no sequence specificity of insertion. *Molecular and General Genetics*.

